# Putamen Atrophy Is a Possible Clinical Evaluation Index for Parkinson’s Disease Using Human Brain Magnetic Resonance Imaging

**DOI:** 10.3390/jimaging8110299

**Published:** 2022-11-02

**Authors:** Keisuke Kinoshita, Takehito Kuge, Yoshie Hara, Kojiro Mekata

**Affiliations:** 1 Department of Rehabilitation, Japanese Red Cross Kobe Hospital, 1-3-1 Wakinohamakaigandori Chuo-ku, Kobe-shi 651-0073, Hyogo-ken, Japan; 2 Department of Neurosurgery, Hyogo Emergency Medical Center, 1-3-1 Hyogokensaigaiiryosenta, Wakinohamakaigandori Chuo-ku, Kobe-shi 651-0073, Hyogo-ken, Japan; 3 Department of Occupational Therapy, Faculty of Rehabilitation, Shijonawate-Gakuen University, 5-11-10, Hojo, Daito-shi 574-0011, Osaka-fu, Japan

**Keywords:** putamen, atrophy, magnetic resonance imaging, functional localization, Parkinson’s disease

## Abstract

Parkinson’s disease is characterized by motor dysfunction caused by functional deterioration of the substantia nigra. Lower putamen volume (i.e., putamen atrophy) may be an important clinical indicator of motor dysfunction and neurological symptoms, such as autonomic dysfunction, in patients with Parkinson’s disease. We proposed and applied a new evaluation method for putamen volume measurement on 31 high-resolution T2-weighted magnetic resonance images from 16 patients with Parkinson’s disease (age, 80.3 ± 7.30 years; seven men, nine women) and 30 such images from 19 control participants (age, 75.1 ± 7.85 years; eleven men, eight women). Putamen atrophy was expressed using a ratio based on the thalamus. The obtained values were used to assess differences between the groups using the Wilcoxon rank-sum test. The intraclass correlation coefficient showed sufficient intra-rater reliability and validity of this method. The Parkinson’s disease group had a significantly lower mean change ratio in the putamen (0.633) than the control group (0.719), suggesting that putamen atrophy may be identified using two-dimensional images. The evaluation method presented in this study may indicate the appearance of motor dysfunction and cognitive decline and could serve as a clinical evaluation index for Parkinson’s disease.

## 1. Introduction

The brain is anatomically divided into the cerebral hemispheres (telencephalon), diencephalon, midbrain, pons, medulla oblongata, cerebellum, and basal ganglia. Each part has functional characteristics. Specifically, the basal ganglia are composed of the striatum, globus pallidus, subthalamic nucleus, and substantia nigra, which are involved in functions such as voluntary movement, eye movements, learning, and memory [1].

The putamen is the portion of the striatum that performs various functions, such as motor control, motivation, emotion regulation, language processing, and memory [2,3,4,5,6]. Although the putamen receives direct input from the ipsilateral hemisphere, its functional properties are unclear. Kumar et al. [7,8] evaluated the function of the putamen by correlating the change in the volume of the dorsal and ventral putamen with motor dysfunction of the oral and upper airway regions in patients with heart failure and obstructive sleep apnea syndrome. In two previous studies, Künzle [9,10] showed that the putamen is primarily involved in motor control and receives projections from the motor and somatosensory areas for that function; Künzle also demonstrated that the motor and somatosensory cortical areas in the upper and lower limbs and trunk, respectively, differentiate where each projection is sent. Various other reports have highlighted the relationship between the function and localization of the putamen [11,12,13,14,15]. However, the putamen is assumed to serve primarily in motor- and emotional regulatory roles, and its functional localization is yet to be fully understood. In our clinical practice, we have observed several patients with putaminal hemorrhage, suggesting that there may be a relationship between the site of putamen damage and physical activity. We consider that the putamen, similar to other brain tissues and nuclei (i.e., thalamus), has a functional localization.

Patients with Parkinson’s disease (PD) are known to have putaminal degeneration. Neurodegenerative diseases, such as PD, are progressive; once they develop, they have a high mortality rate and frequently lead to disability and subsequent lack of independence. Wirdefeldt et al. [16] reported that the incidence of PD is 14–19 per 100,000 cases per year, and the prevalence is estimated to be 100–300 per 100,000 cases. In addition, the 2017 Global Burden of Disease, Injuries, and Risk Factors Study [17] reported that the total number of patients with PD had more than doubled since 1990, indicating that PD is the neurodegenerative disease with the fastest growing prevalence, disability, and mortality. According to the GBD Parkinson’s Disease Collaborators (2018) [18], the factors contributing to the increasing prevalence of PD include aging, longer life expectancy, environmental factors, and decreasing smoking rates. Increased life expectancy in most countries and regions, owing to the development of economic and medical standards, increased the number of older adults, thus, causing an increase in PD incidence and prevalence.

PD is characterized by loss of dopaminergic neurons in the substantia nigra pars compacta, deficiency of dopamine in the striatum, and accumulation of Lewy bodies [19]. In addition, it is associated with motor function disorders, such as tremors, rigidity, and akinesia. These symptoms are caused by hyperexcitability and abnormal inhibition in the basal ganglia, globus pallidus, subthalamic nucleus, and thalamus resulting from degeneration of the substantia nigra pars compacta [20]. PD treatment commonly involves pharmacotherapy, exercise, and functional neurosurgical procedures on the globus pallidus, subthalamic nucleus, and thalamus. Although the globus pallidus and subthalamic nucleus have received the most attention in PD, Santos et al. [21] reported that patients with PD and Parkinson’s syndrome had reduced putamen volumes compared with healthy individuals. Atrophy of the putamen may be an important indicator of the onset of symptoms in PD, such as freezing and short-stepped gait. Therefore, this study aimed to devise a clinically applicable evaluation method for putamen atrophy and to show its relationship with changes in physical function due to PD.

PD is a progressive degenerative disease that is difficult to diagnose, and the clinical diagnosis is based on symptoms. Many diagnostic criteria have been proposed, and various clinical studies have been conducted based on those published by the British Brain Bank [22]. The British Brain Bank diagnostic criteria exclude the possibility of other diseases as much as possible and require patients to have bradykinesia. In addition, the presence of some retentive support factors is a basic diagnostic condition for PD.

Recently, clinicians and researchers have gained more knowledge concerning PD and have sought to develop diagnostic criteria using new testing methods [23]. Futhermore, new diagnostic criteria have been proposed by the International Parkinson and Movement Disorder Society [24]. Therefore, we focused on putamen volume. A previous study used three-dimensional images to investigate volume changes [25]. However, three-dimensional images are mainly used in research; in emergency hospitals that do not have time for initial consultation and clinics that treat outpatients, two-dimensional images from radiography, computed tomography, and magnetic resonance imaging (MRI) are widely used. Therefore, we propose a simple and highly accurate index of putamen atrophy derived from MR images that can be used in clinical practice. Based on this newly proposed parameter, we performed a comparison between patients with and without PD.

## 2. Materials and Methods

### 2.1. Study Design

Thirty-five participants were enrolled in a cooperative medical institution between April 2018 and March 2020. We recruited 16 patients into the PD group and 19 patients into the control group. Patients in the PD group were diagnosed with PD by a neurologist and had no organic disease due to cerebral vascular disease or other neurological diseases. Bilateral MR images from 15 of the 16 patients with PD were available. However, for one patient, the unilateral putamen was difficult to distinguish from the cortex; therefore, only one side was included in the study, resulting in 31 images. Participants in the control group were diagnosed with acute lacunar infarction or transient ischemic attack (TIA) by a neurosurgeon and had no history of organic disease due to cerebral vascular disease or other neurological diseases. For the control group, 30 brain images were included in the study because patients with lacunar infarction had images of the putamen contralateral to the lesion (*n* = 8), and patients with TIA had images of the bilateral putamen (*n* = 11). Both groups were age-matched.

This study conformed to the Code of Ethics of the Declaration of Helsinki and was approved by the ethics committee of our institution (registry number: 502). All participants provided written informed consent for participation in this study.

### 2.2. Imaging Protocol

We used an image acquired after definite diagnosis in the PD group and within 30 min after the onset of lacunar infarction or TIA in the control group. All patients underwent a resting-state MRI examination using a 1.5-T system (Ingenia 1.5T Evolution, Royal Philips, Amsterdam, The Netherlands) equipped with a 12-channel phased-array head coil and a pad on either side of the head to reduce head motion at the time of data acquisition. High-resolution T2-weighted imaging was performed using a magnetization-prepared rapid acquisition gradient-echo pulse sequence (repetition time, 4424 ms; echo time, 100 ms; flip angle, 90°; matrix size, 336; field of view, 230 mm × 184 mm; slice thickness, 5.0 mm; scan mode, MS; number of excitations, 2; water-fat shift, 1.156 pix; and bandwidth, 187.9 Hz). We selected the slices showing the complete thalamus, putamen, and internal capsule. All patients were examined in a relaxed state.

### 2.3. Measurement Method

**Figure 1:**
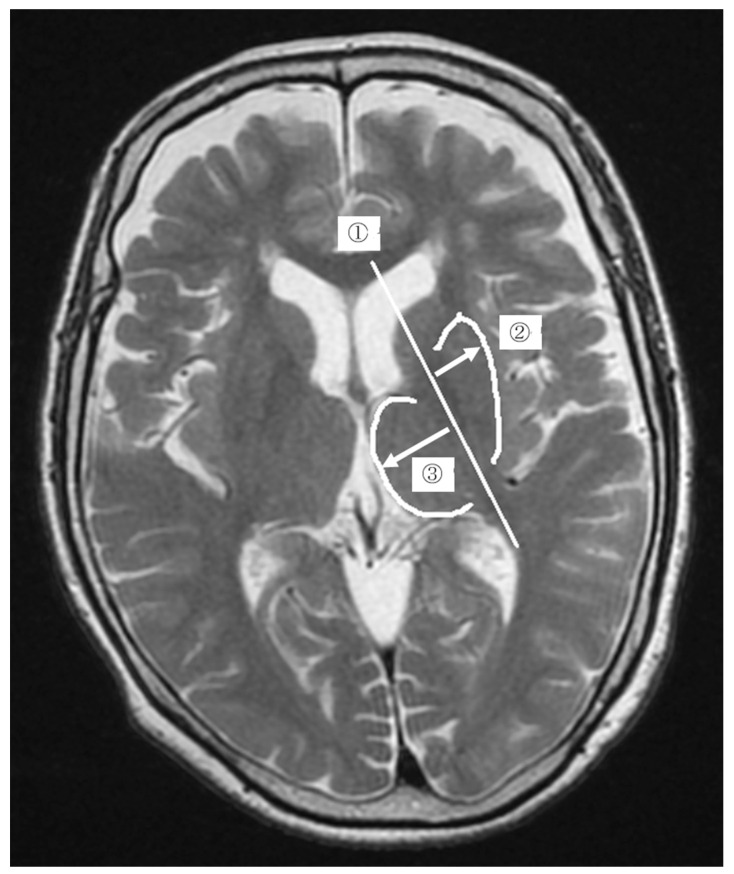
The measurement of the change ratio in the putamen using magnetic resonance images. First, we drew a straight line in the center of the posterior limb of the internal capsule and named it “the posterior limb of the internal capsule line” ➀. Second, we measured the distance from the posterior limb of the internal capsule line to the distal outline of the putamen (Right-pointing white arrow) and named it “putamen distance” ➁. Third, we measured the distance from the posterior limb of the internal capsule line to the distal outline of the thalamus (Left-pointing white arrow) and named it “thalamus distance” ➂. Finally, the ratio of putamen distance to thalamus distance was calculated, and the value was defined as the “change ratio in the putamen”.

We used a built-in application of an electronic medical chart (HOPE/EGMAIN-GX, Fujitsu Limited, Tokyo, Japan) and measured anatomical distances on the images in increments of 0.01 mm. As shown in Figure 1, we defined the distances as the “putamen distance” and “thalamus distance”, respectively. During measurements, the MR images of the PD and control groups were presented randomly to the examiner. Measurements were performed independently by two physiotherapists with 11 and 9 years of clinical experience.

### 2.4. Data Processing

The examiner measured the same region in triplicate for each case, and the average was calculated. Putamen atrophy represents the ratio of the putamen distance to the thalamus distance, and the value was defined as the “change ratio in the putamen”. Both examiners assessed all images, one of whom performed the measurements (two measurements; one on each day).

### 2.5. Statistical Analysis

To determine the reliability of our evaluation method, we analyzed the change ratio in the putamen using the Wilcoxon rank-sum test. The significance threshold was set at *p* < 0.05. Intraclass correlation coefficients (ICCs) were calculated using the SPSS Statistics software program version 27.00 (IBM Corp., Armonk, NY, USA). A good ICC was considered to be ≥0.7.

## 3. Results

### 3.1. Demographics

The demographic data of the 16 and 19 patients in the PD and control groups, respectively, are as follows: the PD group comprised seven men and nine women, and the control group comprised 11 men and eight women. The mean age of the patients in the PD group was 80.3 ± 7.30 years, and that of the control group was 75.1 ± 7.85 years; there was no significant difference in age between the groups.

### 3.2. Change Ratio in the Putamen

**Figure 2:**
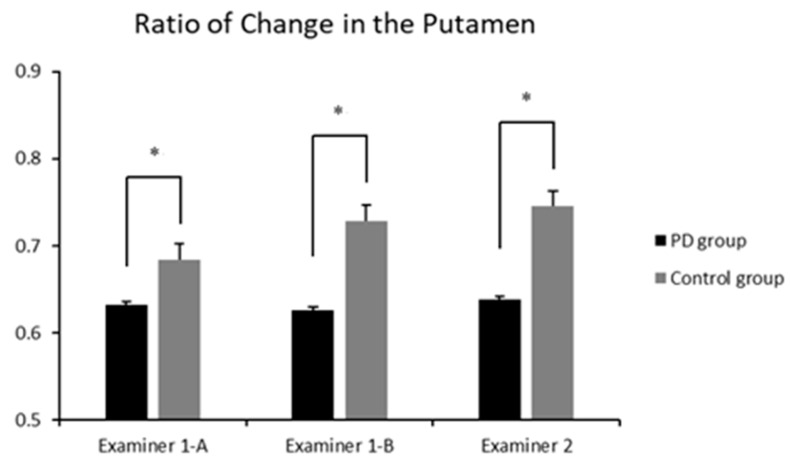
Average change ratio in the putamen for each group. The number represents the putamen size for a thalamus size equal to 1. Error bars indicate standard deviation. * *p* < 0.05.

The average change ratio in the putamen calculated from the measured values is shown in Figure 2.

For the first measurement by Examiner 1, the average change ratio in the PD group was 0.632 ± 0.067, and that of the control group was 0.684 ± 0.082 (Figure 2, Examiner 1-A; *p* = 0.017, F = 2.38). For the second measurement on a different day by Examiner 1, the average change ratio in the PD group was 0.626 ± 0.064, and that of the control group was 0.728 ± 0.077 (Figure 2, Examiner 1-B; *p* = 0.0019, F = 4.76). For Examiner 2’s measurement, the average change ratio in the PD group was 0.638 ± 0.095, and that of the control group was 0.745 ± 0.114 (Figure 2, Examiner 2; *p* = 0.0028, F = 3.63). Thus, there were significant differences between the groups. The overall average change ratio in the PD group was 0.633 ± 0.079, and that of the control group was 0.719 ± 0.096.

Comparing the left and right sides, the first measurement by Examiner 1 showed that the average change ratios in the right and left sides of the PD group were 0.644 ± 0.067 and 0.619 ± 0.065 (*p* > 0.10, F = 1.10), respectively. The second measurement on a different day by Examiner 1 showed that the average change ratios in the right and left sides of the PD group were 0.640 ± 0.056 and 0.611 ± 0.069 (*p* > 0.10, F = 1.34), respectively. Examiner 2’s measurement showed that the average change ratios on the right and left sides of the PD group were 0.653 ± 0.065 and 0.622 ± 0.011 (*p* > 0.10, F = 1.30), respectively. There was no significant difference between the measurement results of the left and right sides.

### 3.3. Intraclass Correlation Coefficients

The ICC for intra-rater reliability was 0.842 (95% confidence interval: 0.676–0.924).

## 4. Discussion

### 4.1. Overview

This study aimed to propose and validate a method for evaluating putamen atrophy using MRI and investigate whether the change ratio in the putamen corresponds to PD by associating it with PD changes. Our results showed that this evaluation method had sufficient intra-observer reliability and a small intra-examiner measurement error. Furthermore, the change ratio in the putamen varied significantly between the PD and control groups, suggesting that it corresponds to the diagnosis of PD.

### 4.2. Method for the Evaluation of Putamen Atrophy

In this study, we proposed to use a change ratio determined from MR images to evaluate putamen atrophy. The ICC showed satisfactory intra-rater reliability of 0.842. Currently, MRI allows three-dimensional brain image processing to be performed through methods such as MRIcron (https://www.nitrc.org/projects/mricron, accessed on 20 August 2020) and generalized auto-calibrating partially parallel acquisitions [26]. However, three-dimensional images are not yet widely used in emergency hospitals and initial medical examinations because more time is required for image processing. In contrast, two-dimensional images are widely used in clinical practice because they are less time-consuming and less burdensome for the patient; therefore, they offer significant advantages during the initial examination. The evaluation method presented here is simple and can be evaluated by any medical professional in the field. Furthermore, three-dimensional images take time to analyze and are frequently used in research. This evaluation method is quick and simple as it can be performed from MRI and used as a parameter for screening and diagnosis of PD.

In addition, there are no reports of changes in the thalamus volume, although there are scattered reports of changes in the putamen volume in PD [27]. Therefore, the change ratio in the putamen was calculated for that in the thalamus. There has been much discussion regarding intra- and inter-examiner errors in diagnostic imaging for some time, and there is a need for clear protocols for diagnostic imaging [28,29,30]. As measurements are performed through visual evaluation of the posterior limb of the internal capsule, thalamus, and putamen, image discrimination is influenced by reader experience. However, here, the ICC was >0.7 and showed reproducibility, with a small error for the same examiner. The same examiner can conduct evaluations on different days, and no difference will be observed in the evaluation results, making its usage highly valuable. Although individual differences can affect putamen measurements, clinicians can compensate for potential errors using the ratio with the thalamus distance. Atrophy of the capsule occurs at various locations [8], and capturing three-dimensional changes in two dimensions is challenging. However, this study’s proposed evaluation method results showed significant differences between the two groups. We believe that the method captures changes in the position of the nucleus owing to changes in its external shape and atrophy. Hence, comparing the change ratio in the putamen could capture volumetric changes or external shapes in the putamen.

### 4.3. Change in Ratio in the Putamen in Both Groups

A significant difference in the change ratio in the putamen was observed between the PD and control groups (*p* = 0.015). Our results suggest a difference in the change ratio in the putamen between patients with and without PD and PD, which may reflect putamen atrophy. PD can be considered to cause changes in the putamen [31,32]. However, it is important to note that aging can also lead to putamen atrophy [33]. Structural neuroimaging studies applying a longitudinal design have shown that global and regional gray matter volume declines with age in healthy individuals [34,35,36]. Thambisetty investigated longitudinal changes in cortical thickness in 66 healthy older adults aged 60–84 years [37]. The study showed that age-related decline in cortical thickness is widespread, and an anterior–posterior gradient with frontal and parietal regions exhibits greater rates of decline than temporal and occipital regions. There were no significant differences between the two groups of patients in this study. However, previous studies have commonly evaluated them separated by decades; therefore, the 5-year difference and degree of age-related atrophy remain debatable. Further comparison of the left-right difference showed that the left side changed more, although there was no significant difference. These results are consistent with those of previous studies, showing that the left side is predominantly impaired [38,39,40].

Patients with lacunar infarction or TIA were grouped as a control. The control group used MRI images within 30 min of onset. Muhammad et al. reported no significant difference in age-related brain atrophy at 18 months after TIA compared with normal participants. Therefore, the MRIs used in this study were obtained within 30 min of TIA onset and are not expected to be affected by lesion-induced brain atrophy. We also used images on the opposite side of the lacunar infarct from the impaired side, which we did not expect to be affected by lesion-induced brain atrophy.

In healthy individuals, PD or multiple system atrophy does not occur; therefore, faster putamen atrophy than that associated with aging might predict the appearance of movement disorders, such as frozen gait and brachybasia. Therefore, using the change ratio in the putamen as an indicator of PD may reduce the risk of developing the disease. In addition, connecting patients to early intervention might prevent PD-associated decline in activities of daily living and quality of life and extend healthy life expectancy.

### 4.4. Limitations

This study had some limitations. First, the image processing accuracy between the examined images was similar because the MR images used in this evaluation were acquired at the same institution using the same imaging protocol. However, the impact of equipment or imaging protocol differences on image assessment accuracy is yet to be evaluated. Therefore, in the future, it will be necessary to compare the results using MR images obtained under varying conditions. Furthermore, changes in volume should be confirmed by 3D imaging. There are various brain volumetric measurements using 3D imaging, each with its own characteristics [41]. The use of 3D images is mainly for research, and there are issues to be resolved before they can be applied in clinical practice. The evaluation method presented here is simple because it uses MRI and can be used as a diagnostic parameter. Based on these factors, a future issue is to compare and examine the values of the ratios calculated using the volumes of the putamen and thalamus obtained using 3D images with the results of this study using 2D images.

Second, the internal capsule, putamen, and thalamus positions on MR images were used for the evaluations. Such evaluations require a high degree of reader experience and high-quality images to distinguish between the internal capsule, putamen, and thalamus. Precisely, these measurements evaluate the lentiform nucleus of the putamen and globus pallidus. According to Matochik et al. [42], the volume loss in the globus pallidus, which has less dopaminergic input than the striatum, should be small. We adopted this method because we speculated that it would lead to the evaluation of the putamen by assessing changes in the lentiform nucleus; however, there is still room for improvement.

Finally, the patient sample was small, and we focused on patients with PD as a group with potential putamen atrophy. Multiple system atrophy is a disease associated with putamen degeneration. Mainly, striatonigral degeneration is caused by degeneration of the putamen, of which Parkinsonism is a typical symptom. We would like to improve the accuracy of this diagnostic marker by increasing the number of cases, examining the evaluation method, and determining the cutoff value of the rate of change in the putamen for PD. In the future, we would like to improve the accuracy of the proposed evaluation method by improving the classification of patients in the PD group according to years of treatment and disease severity and by including patients with striatal substantia nigra degeneration to improve our understanding of the role of the putamen in this neurodegenerative disease.

## 5. Conclusions

Here, we proposed a method to evaluate putamen atrophy using MRI. The change ratio in the putamen was significantly lower in patients with PD than in controls, suggesting that it represents putamen atrophy. In addition, this method could be applied for early detection of PD and preventive medicine by improving the accuracy in measuring the change ratio in the putamen.

## Data Availability

Study data are available from the corresponding author on reasonable request.
